# Enhancing the Therapeutic Potential of Olfactory Ensheathing Cells in Spinal Cord Repair Using Neurotrophins

**DOI:** 10.1177/0963689718759472

**Published:** 2018-05-31

**Authors:** A. A. Wright, M. Todorovic, J. Tello-Velasquez, A. J. Rayfield, J. A. St John, J. A. Ekberg

**Affiliations:** 1 Clem Jones Centre for Neurobiology and Stem Cell Research, Griffith Institute for Drug Discovery, Griffith University, Nathan, Queensland, Australia; 2 Menzies Health Institute Queensland, Griffith University, Southport, Queensland, Australia

**Keywords:** autologous transplantation, glia, growth factors, cell proliferation, neuron

## Abstract

Autologous olfactory ensheathing cell (OEC) transplantation is a promising therapy for spinal cord injury; however, the efficacy varies between trials in both animals and humans. The main reason for this variability is that the purity and phenotype of the transplanted cells differs between studies. OECs are susceptible to modulation with neurotrophic factors, and thus, neurotrophins can be used to manipulate the transplanted cells into an optimal, consistent phenotype. OEC transplantation can be divided into 3 phases: (1) cell preparation, (2) cell administration, and (3) continuous support to the transplanted cells in situ. The ideal behaviour of OECs differs between these 3 phases; in the cell preparation phase, rapid cell expansion is desirable to decrease the time between damage and transplantation. In the cell administration phase, OEC survival and integration at the injury site, in particular migration into the glial scar, are the most critical factors, along with OEC-mediated phagocytosis of cellular debris. Finally, continuous support needs to be provided to the transplantation site to promote survival of both transplanted cells and endogenous cells within injury site and to promote long-term integration of the transplanted cells and angiogenesis. In this review, we define the 3 phases of OEC transplantation into the injured spinal cord and the optimal cell behaviors required for each phase. Optimising functional outcomes of OEC transplantation can be achieved by modulation of cell behaviours with neurotrophins. We identify the key growth factors that exhibit the strongest potential for optimizing the OEC phenotype required for each phase.

## Introduction

Spinal cord injury (SCI) can lead to permanent damage for which there is currently no cure. SCI causes damage to neural tissue, initially due to the direct trauma, which then progresses due to a series of secondary cellular events causing further damage. After injury, local inflammation, ischemia, and oxidative stress result in expansive cell death and damage at the SCI site^1^. Subsequently, reactive astrocytes undergo hypertrophy, proliferate, and migrate to the injury site. They then create a glial scar that impedes growth and reinnervation of neurons in this area and which acts as a tertiary lesion^1–4^.

**Fig. 1.**
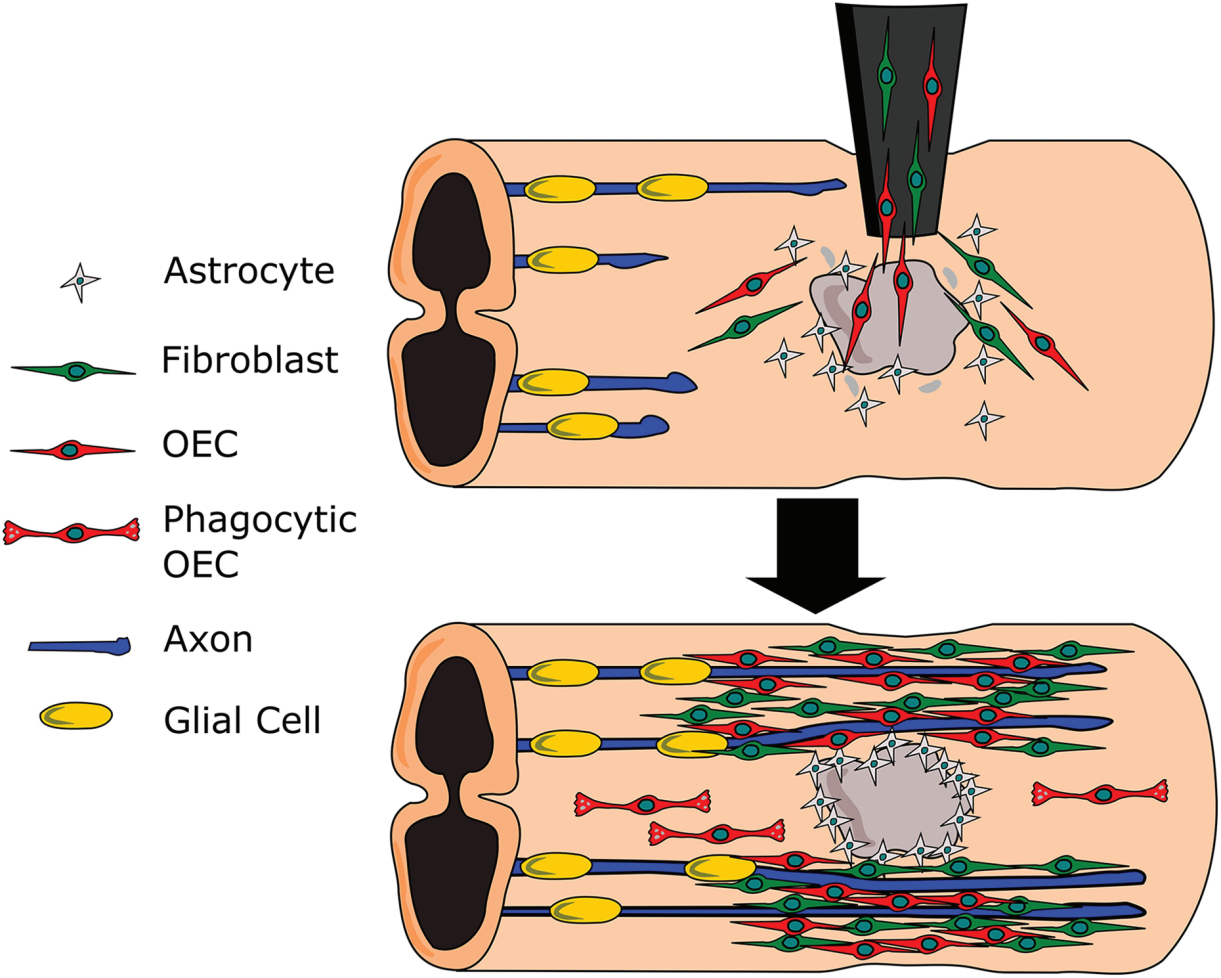
Olfactory ensheathing cells (OECs) and fibroblasts administered to a Schwann cell site (gray). The mixed cell culture supports and ensheathes the regenerating axons. OEC phagocytose scar and damaged tissues.

A promising therapy for SCI is the autologous transplantation of olfactory ensheathing cells (OECs), the glial cells of the primary olfactory nervous system. OECs are taken from the olfactory epithelium of the nasal cavity, cultured in vitro, and then transplanted into the damaged SCI site (Fig. 1)^5^. OECs are present in the primary olfactory nervous system, which comprises the olfactory nerve and the nerve fiber layer (NFL) of the olfactory bulb (OB). OECs naturally promote the continuous regeneration of the olfactory nerve that occurs throughout life and therefore exhibit unique growth-promoting properties. OECs are also capable of migrating long distances into and interacting with astrocytic glial scar tissue^3^, as well as with other cells that may be present in the injury site^6^, resulting in a 3-dimensional framework conducive to axonal extension. This developing treatment has been trialed in rats, dogs, and humans, where it has been shown to be safe and capable of promoting functional repair in the form of motor and sensory innervation and allowing for weight bearing movement to varying levels of success^7–11^. However, in order to create a therapeutic intervention capable of providing consistent results, autologous OEC transplant therapies must be improved.

There are many reasons why outcomes of OEC intervention for spinal cord repair vary from trial to trial. There are several broadly different methods for inducing SCI in animal models including hemisection, transection, and contusion injuries, which all have different effects on the extent of injury. The injuries can all be performed at various cervical and thoracic levels which again lead to variations in outcomes of the OEC intervention. With respect to the use of OECs themselves, discrepancies between preclinical trial results can be broadly attributed to (1) exact anatomical source of the OECs (different subpopulations of OECs exist with distinct biological properties^12^), (2) OEC purity, and (3) OEC survival rates after transplantation.

As a first step toward improving outcomes and consistency in the use of OECs, the purification, survival, and behavior of OECs could be optimized for transplantation. One proposed method of achieving reproducibility is by using neurotrophins, for which OECs express a multitude of receptors, to promote OEC survival and to drive the cellular behaviors into those most favorable for transplantation and regeneration.

A number of studies have highlighted the potential of exploiting OEC characteristics for therapeutic use within the injured spinal cord. Within the olfactory system environment, OECs aid and support the extension of axons from olfactory sensory neurons which reside in the olfactory mucosa in the peripheral nervous system (PNS). The OECs guide the axons up to the OB within the central nervous system (CNS) where the OECs then contribute to the sorting of olfactory axons to their appropriate targets within the olfactory bulbar glomeruli as dictated by the olfactory receptor profile of the neurons^13–15^. Furthermore, OECs can infiltrate scar tissue and astrocytic environments. Due to their unique heparin profile^3^, OECs can interact and intermingle with astrocytes in ways that Schwann cells (SCs) and oligodendrocytes cannot. This feature may allow OECs to disperse throughout the glial scar which is formed following SCI, providing a scaffold for neuronal growth through an otherwise neurotoxic environment. Furthermore, OECs have been shown to possess phagocytic capabilities^16,17^. In the olfactory system, this capability allows for the removal of axonal debris during early development as well as a mechanism for bacterial removal^18^; however, within the injured spinal cord, this may allow for removal of cytotoxic debris that can inhibit the regeneration of neurons. Further studies into OEC properties have demonstrated facilitation of axon extension^19^, secretion of neuroprotective and stimulatory proteins which can enhance the properties of neurons and other glia^20,21^, and an ability to integrate and support other cell types^22^. Within the context of SCI, these cellular behaviors may prove ideal for supporting and guiding axonal regeneration to reform severed connections. However, what is now needed is to further optimize these features to enhance the therapeutic potential of OECs and to achieve consistency between treatments.

OECs express a variety of neurotrophin receptors including p75 neurotrophin receptor (P75NTR); tropomyosin receptor kinase A (TrkA), tropomyosin receptor kinase B (TrkB), and tropomyosin receptor kinase C (TrkC); 2 types of glial-derived neurotrophic factor (GDNF) family receptors: GDNF receptor alpha 1 (GFRα-1) and the tyrosine kinase receptor RET; and the fibroblast growth factor (FGF) receptor 1 (FGFR1; Table 1). Neurotrophins acting on these receptors have been shown to promote favorable OEC behaviors, in particular cell migration and cell–cell interactions^19,37^, as well as influence the survival and viability of neurons and their axonal extensions^38,39^. Additionally, many of these receptors are also present on neurons, oligodendrocytes, SCs, astrocytes, and other supporting cells found throughout the nervous system. Using neurotrophins as therapeutic agents may not only be utilized to target transplanted OECs but also to aid in the regeneration of neurons at the injury site—a process involving modulation of these other cell types.

**Table 1.** Receptor Expression and Corresponding Cell Types.

Neurotrophin Receptor	Cell Type	Neurotrophin	References
TrkA	Neurons	NGF and NT-3	^23^
TrkB	Neurons and OECs	BDNF, NT-3, and NT-4	^23^
TrkC	SCs, neurons, and OECs	NT-3	^23,24^
P75NTR	SCs, OECs, and dorsal root ganglion neurons	BDNF and NGF	^24–26^
GFRα-1	SCs, OECs, and neurons	GDNF	^27–30^
RET	OECs and neurons	GDNF	^29,30^
VEGFR2	Blood vessel endothelial cells	VEGF	^31,32^
EGFR	Activated astrocytes and oligodendrocytes	EGF	^33,34^
FGFR1	Astrocytes, OEC, SCs, and fibroblasts	FGF2	^3,35^
PDGFRα	Fibroblasts, oligodendrocytes, and astrocytes	PDGF-AA and PDGF-BB	^36^

Abbreviations: TrkA, tropomyosin receptor kinase A; TrkB, tropomyosin receptor kinase B; TrkC, tropomyosin receptor kinase C; P75NTR, p75 neurotrophin receptor; GFRα-1, GDNF receptor alpha 1; RET, receptor tyrosine kinase; PDGF, platelet-derived growth factor; PDGFR, PDGF receptor; OEC, olfactory ensheathing cell; SCs, Schwann cells; NGF, nerve growth factor; BDNF, brain-derived neurotrophic factor; NT, neurotrophin; GDNF, glial-derived neurotrophic factor; VEGF, vascular endothelial growth factor; VEGFR2, VEGF receptor 2; EGF, epidermal growth factor; FGF2, fibroblast growth factor 2; PDGF-AA, dual alpha-subunit of PDGF; PDGF-BB, dual β-subunit of PDGF.

## Using Neurotrophins as Therapeutic Agents in SCI Repair

Using OECs to repair the injured spinal cord involves (1) harvesting and expansion of OECs, then (2) transplantation followed by (3) integration and survival of the cells at the site of injury^40^. All 3 phases must be successful to achieve a significant functional outcome. Through the use of neurotrophins, it may be possible to enhance the outcomes of all 3 of these phases, with a combination of neurotrophins being tailored to the requirements of each phase. Thus, neurotrophins can (1) be applied to OECs following biopsy to promote proliferation in preparation for transplantation, (2) be coadministered along with OECs to support the cells during transplantation, and (3) act in the longer term at the SCI site, leading to greater integration and survival of OECs following transplantation. Within the final phase, the neurotrophins may also exert important effects on other cell types, such as cell–cell interactions or remyelination, as well as on the extracellular environment, for example, altered growth factor profile of the extracellular matrix.

## OEC Proliferation and Preparation Prior to Transplantation into the Injured Spinal Cord

Key factors accounting for the variable outcomes of OEC transplantation are the quality, purity, and source of the cells—all of which may influence the phenotype and behavior of the OECs to be transplanted. A high-quality population of OECs must be prepared prior to application and should consist of cells that are (1) highly proliferative, to achieve an appropriate number of cells for transplantation in a prompt time period, allowing the transplantation to occur sooner, and (2) resilient to surviving the transplantation process—thus increasing the number of viable cells transplanted. It may be possible to manipulate this preparatory period using neurotrophins to adjust OEC phenotype, induce purification, and improve viability of OEC populations.

### OEC Source and Phenotype

**Fig. 2.**
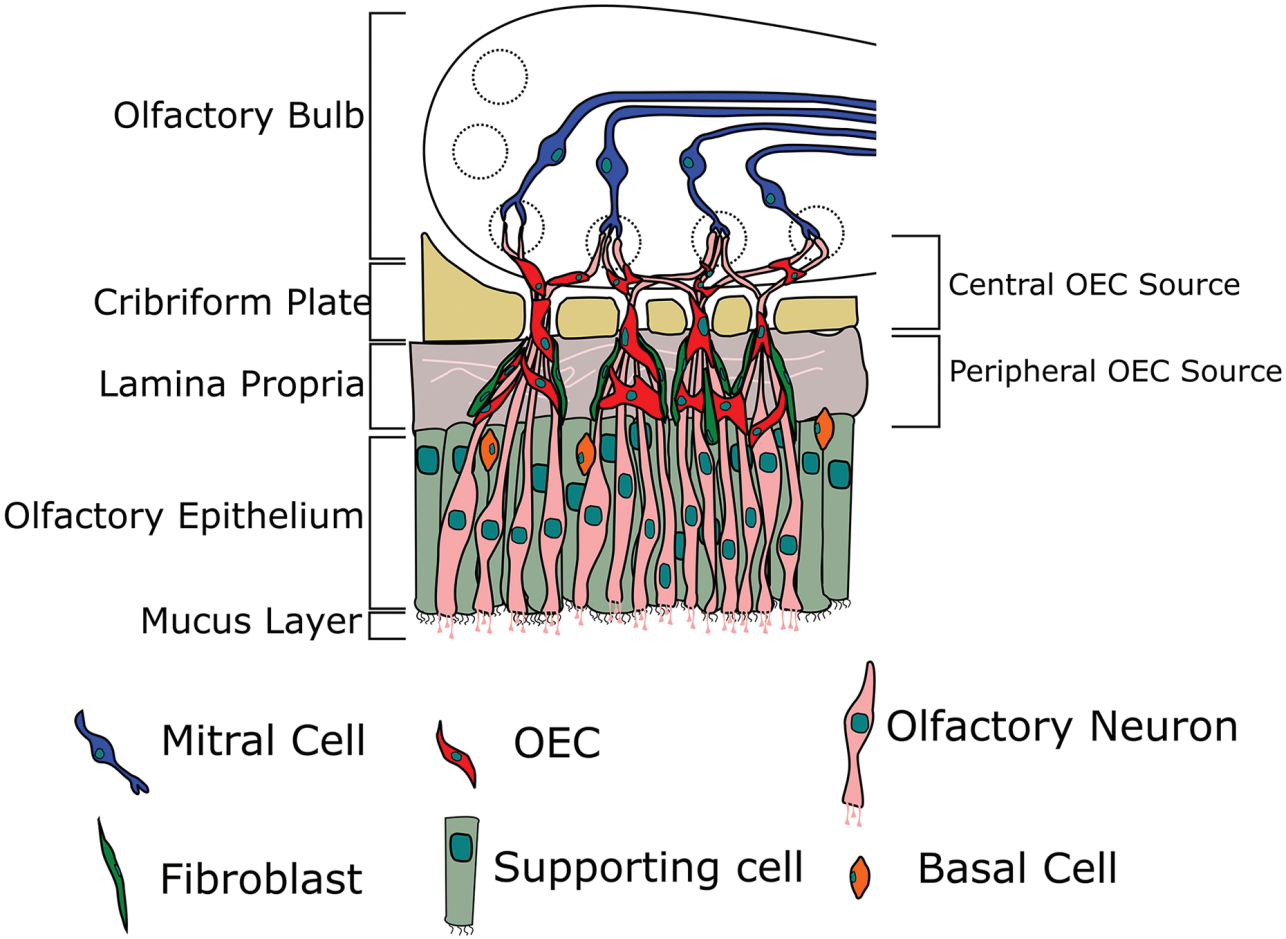
Location of olfactory ensheathing cells (OECs) within the olfactory system. Olfactory neurons extend axons from the nasal cavity through the cribriform plate and make connections within the glomeruli with mitral cells. OECs ensheathe the olfactory axons, aiding their growth and directing them to form their connections.

OECs are present throughout the entire olfactory nervous system from inside the nasal cavity to the OB^14^. OECs exhibit distinct roles based on their location and thus different phenotypes. OECs within the lamina propria (LP-OECs) of the olfactory mucosa are located along the length of the olfactory nerve and guide axons from olfactory sensory neurons as they project toward the OB. The OECs create channels through which the axons extend, and the channels formed by OECs are in turn encompassed by fibroblasts that act as a perineurium-like structure^41^. In contrast to SCs (the main glia of the PNS), OECs do not myelinate individual axons but rather ensheathe small fascicles (bundles) of unmyelinated axons in a process similar to the early stages of nervous system development^42^. OEC channels guide the immature olfactory sensory neuron axons through the cribriform plate into the NFL of the OB in the CNS. OECs within the NFL of the OB-OECs have characteristics different from that of the LP-OECs as they aid the sorting and directing of axons toward their appropriate glomerulus according to their respective odorant receptor (Fig. 2), for which there are 400 functionally distinct genes in humans^13,14^.

Both LP-OECs and OB-OECs have been used for SCI transplantation studies of animals and humans. A recent study showed that transplanting OB-OECs from the OB produced functional recovery in a 38-y-old SCI patient who had a spinal cord transection leaving an 8-mm gap in the spinal cord at thoracic vertebral level nine^10^. While the transplantation resulted in improved motor and sensory functional outcomes in this case, harvesting cells from the OB can cause severe side effects, such as hyposmia and depression^43^. For this reason, it is favorable to use a less invasive biopsy procedure to obtain LP-OECs from the olfactory mucosa^5,44^. It is, however, possible that OECs from distinct anatomical sources may exhibit differential ability to promote axonal regeneration. One review systematically compared multimetric outcomes (electrophysiological, behavioral, and magnetic resonance imaging results) between LP-OECs and OB-OECs and found that the 2 cell types induced comparable outcomes; however, the advantage of OB-OECs was that they do not need the same extent of purification as peripheral OECs^45^. Thus, both peripheral and bulb OECs exhibit advantages and disadvantages. If future studies determine that OB-OECs indeed do lead to better outcomes than LP-OECs, it is also possible that growth factor modulation can change the phenotype of LP-OECs toward a type with similar features to OB-OECs. Therefore, a thorough characterization of how growth factors modulate OECs is highly warranted in the field.

### Cell Purity

Another variability between studies is the purity of the OECs. As the common biopsy region also contains fibroblasts and other contaminant cells, there have been studies focusing on purification methods for OECs^46^. Currently, the importance of OEC population purity for transplantation is debated, as other cell types from the olfactory epithelium may also exert important beneficial effects on neural regeneration^7,10,47,48^. In the previously described case showing functional improvement of a patient with thoracic level 9 SCI, OECs comprised only 16% of the cells transplanted with the majority of cells likely being fibroblasts^10^. Additionally, pure OECs have been suggested to be less resilient to the harsh environment of the SCI site^10^. However, regardless of whether a homogenous or heterogeneous OEC population is more beneficial, purifying these populations is essential so that the optimal ratio between OECs and other cell types, in particular fibroblasts, can be determined.

**Fig. 3.**
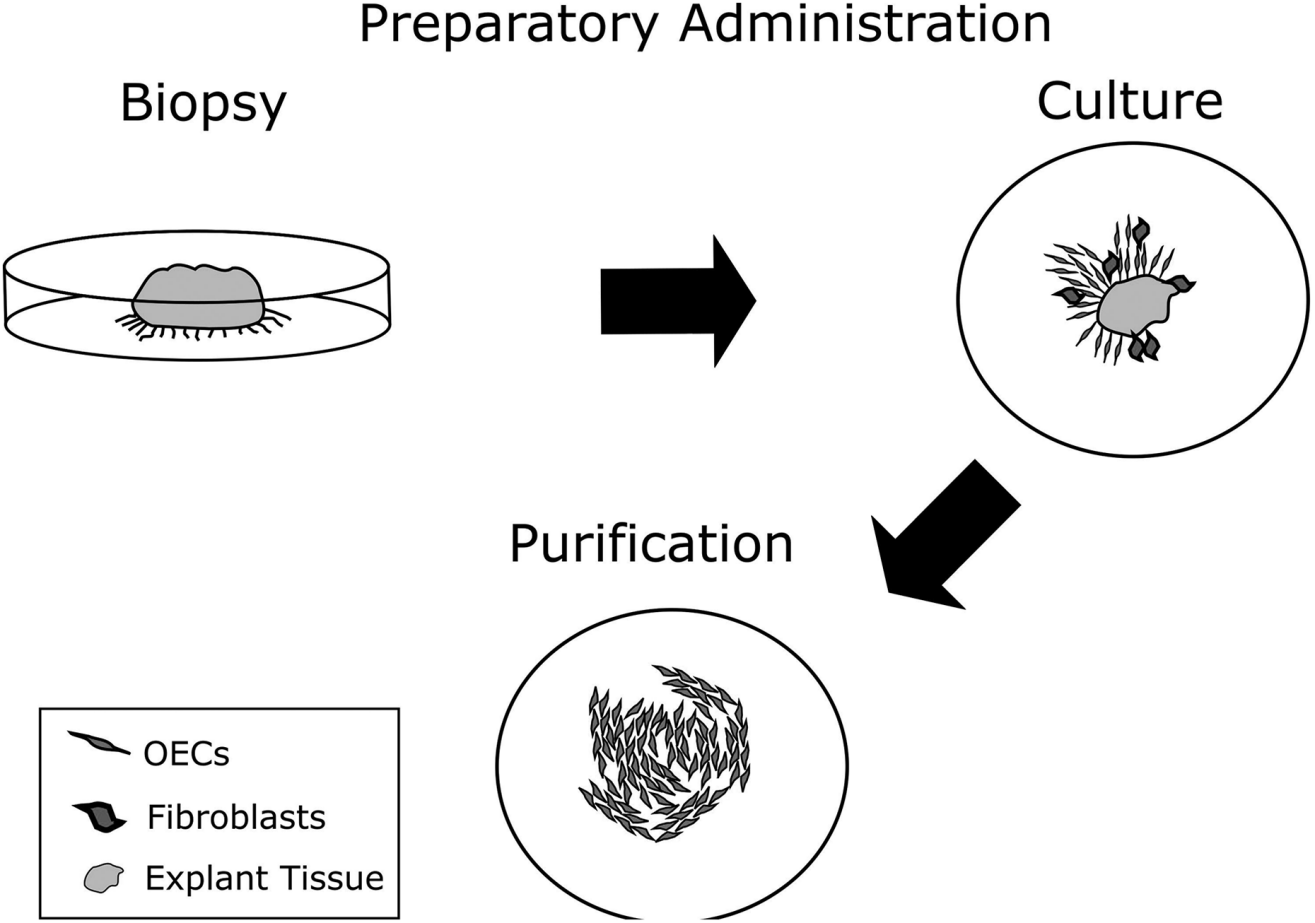
Primary olfactory ensheathing cells (OECs) are cultured from an explant tissue. Following the initial outgrowth, the explant is removed and the cells are passaged. Neurotrophin 3 may be used as a purification agent to facilitate expansion of OECs rather than fibroblasts^8^.

The aforementioned issues may be addressed using neurotrophins by favoring OEC purity through selective promotion of proliferation and by improving resilience of cells through neuroprotective effects. Neurotrophin-3 (NT-3) is currently used as a purification agent and has been shown to stimulate proliferation of OECs over other cells (Fig. 3). This method has been demonstrated to achieve a 90% to 95% pure population of OECs by passage 5^46^. Furthermore, human OECs cultured in the presence of NT-3, demonstrated a 25% increase in cells that fit OEC receptor expression and appearance, and a 30% decrease in cells that did not^46^.

### Cell Viability

An important point to consider is whether supporting the cells with neurotrophins prior to transplantation can improve the long-term quality of the OECs after transplantation. While OECs may thrive in vitro, following transplantation, around 99% of the transplanted cells die^49^. This issue may also be addressed by manipulating the cells with neurotrophins in culture to improve cellular resilience to pathological and noxious environments following transplantation. To date, relatively little is known regarding which neurotrophins enhance the viability of OECs. NT-3 is likely to aid in this process as it promotes proliferation and survival of OECs (discussed earlier). NT-3 also exerts beneficial effects on other neural cells; for example, it has been shown to combat apoptosis and prolong survival of chronically injured neurons when applied locally to an SCI site together with OECs^50^. Additionally, OECs transfected to express higher levels of NT-3 have been shown to induce a much greater recovery in an injured rat model compared to NT-3 or OECs alone^51^. While it is possible that NT-3 has a direct effect on OEC viability, this has not been proven experimentally to date.

In addition to NT-3 and GDNF, OECs secrete nerve growth factor (NGF) and brain-derived neurotrophic factor (BDNF). LP-OECs and OB-OECs from the outer NFL express the NGF receptor P75, along with TrkA, TrkB, and TrkC which are receptors for NGF, BDNF, and NT-3, respectively^52–54^. Thus, while NGF may exert a regulatory role on OECs, the OEC features and behaviors regulated by NGF remain unknown. NGF is known to exert a neuroprotective role on other nervous system cells, for example, inhibiting apoptosis in a neural cell line (PC12 cells) via binding to the TrkA receptor^55^. Conversely, while NGF has been demonstrated to be neuroprotective, it has also been shown to play a role in the stimulation of apoptosis in other cell types. This variable effect is due to the differential binding affinities of the Trk receptors and P75NTR. For example, NGF, BDNF, and NT-3 bind to TrkA, TrkB, and TrkC, respectively, but also have low-affinity binding with P75NTR. P75NTR has been linked with effects of cell survival, apoptosis, proliferation, differentiation, regeneration, axonal extension, and neuronal outgrowth depending on the cells that express them^56–59^. The range of pathways and cobinding involving these receptors can lead to opposing effects depending on several factors including cell health, ceramide presence, neurotrophin concentration, and others^4,59,60^. To date, the effects of growth factors on OECs, in particular on different OEC populations, remain to be determined. In summary, NT-3, NGF, GDNF, and BDNF may all play important regulatory roles relating to OEC viability, but this remains to be systematically determined.

## Coadministration of Neurotrophins during OEC Transplantation

Although cell quality may be improved by pretreatment with neurotrophins, neurotrophic factors can also be coadministered during transplantation. This can address the issue of cell death primarily in 2 ways: (1) by stimulating protective pathways preventing cell death and (2) by stimulating cell proliferation to promote cell replenishment. Furthermore, neurotrophin stimulation can promote cell–cell interactions, resulting in better cell survival, contact-mediated migration, and integration^6^. The successful use of neurotrophins during the transplantation process should aid in the increase of the cellular resilience, decrease in the cytotoxic environment, and promote the successful integration of the transplanted cells.

### Neuroprotection during Transplantation

NGF would appear to be a possible candidate for coadministration with OECs. NGF exhibits protective effects against TrkA-positive cells such as neurons and may be able to further support neurons in addition to the effects of OECs^55^. However, NGF binding to P75NTR during stress stimulates a stress-related pathway. In this pathway, ceramide, (a lipid molecule found in the cell membrane), phosphorylates the stress-activated protein kinase (SAPK), the downstream effect of which is cell death. Therefore, NT-3 may be a more appropriate candidate as it has demonstrated clear effects on OECs that are likely to be beneficial in vivo^46,55^.

### Phagocytosis during Transplantation

As mentioned previously, following cell transplantation, most cells will not survive^49^. In addition to being counterproductive to transplantation efforts, this cell death leaves a large swath of toxic cellular debris within the transplantation site, which can be detrimental to neuron growth. One aspect of glial cell biology that has only recently been characterized is glial-mediated phagocytosis. Phagocytosis of axonal debris is a crucial aspect of the continuous regeneration that occurs in the olfactory nerve, and it is a property of OECs that may be particularly important in spinal cord regeneration^61^. OECs are highly phagocytic^61^, and so by engulfing this cellular debris, OECs create an environment more favorable for axonal regeneration. Increasing the phagocytic capability of OECs may therefore be highly beneficial for functional outcomes following transplantation, and thus identifying neurotrophins that enhance this effect is highly warranted.

To date, the effects of neurotrophins on phagocytosis are largely unknown. Natural products have been utilized to increase phagocytic potential of OECs, which is highly correlated with increased activity of lamellipodial (membranous) extensions^62^. While the direct effect on phagocytosis by growth factors is not yet characterized, neurotrophins capable of inducing lamellipodia, such as GDNF^6^, may also enhance the phagocytic potential of OECs. Additionally, NGF and BDNF play a neuroprotective role and can cause behavioral changes in OECs via the Mitogen-activated protein kinase kinase / extracellular signal-related kinase (Mek/Erk) pathways^63^ which also regulate phagocytic activity^62^. While the possibility of these neurotrophins influencing the phagocytic activity of OECs has not been investigated, they present a possible starting point for research into using neurotrophins to manipulate OECs into a state that is conducive for consistent functional SCI repair.

### Modulation of the Inflammatory Response within the SCI Site by Transplanted OECs

In addition to phagocytosing debris, which may aid in clearing the injury site, OECs may modulate the endogenous inflammatory response (mainly mediated by astrocytes and microglia) in a manner favoring neural regeneration. Microarray analyses have shown that OECs express a large number of cytokines involved in the recruitment of immune cells^64–66^, suggesting that OECs do indeed interact with immune cells and regulate inflammatory responses. After injury (bulbectomy), OECs upregulate expression of interleukin 6 and its receptor, which may play a key role both in mediated axonal regeneration and in modulation of inflammation^67,68^.

The presence of OECs does not induce stress responses in astrocytes^69^. In fact, the presence of OECs reduced the expression of glial fibrillary acidic protein (GFAP; a marker of reactive astrocytes) in an in vitro model of astrogliosis^70^. One study compared the expression of proinflammatory cytokines and markers of astrocyte reactivity between OEC-transplanted and mock-transplanted rats with SCI. OEC-transplanted rats exhibited an earlier peak in the immunoreactivity of inducible nitric oxide synthase, GFAP (a marker of reactive astrocytes), and tomato lectin (a marker of microglia) than mock-transplanted animals, suggesting that one of the mechanisms by which OECs are neuroprotective is by causing an earlier, shorter immune response by astrocytes and microglia^71^. OECs have also been shown to attenuate stimulated nuclear factor κβ translocation in astrocytes, a key response involved in astrocyte activation/inflammation, along with expression of the proinflammatory cytokine granulocyte macrophage colony-stimulating factor. It is thought that insulin-like growth factor-1 secreted by OECs contributes to this modulation of astrocyte activation^72^.

Thus far, the mechanisms by which OECs may modulate the inflammatory state and activation of astrocytes and microglia after transplantation into the injured cord remain a largely unexplored field. In particular, it remains unknown how neurotrophins regulate OEC-mediated modulation of inflammation. The astrocyte scar is detrimental for axonal regeneration, and thus a characterization of how OECs modulate inflammatory cells within the injury site may lead to significantly enhanced therapeutic potential of transplanted OECs.

### Cell Transplant Integration

A successful therapeutic outcome following OEC transplantation into an SCI site is highly dependent on the ability of the transplanted cells to integrate into the transplantation site. OECs have shown to rely upon cell–cell contact for growth stimulation, and so a neurotrophin that encourages this action would be beneficial for integration of the transplanted cells within the SCI site. GDNF is an interesting candidate with already proven beneficial effects on OECs, such as membrane protrusions (so-called lamellipodial waves) that promote cell–cell contacts, resulting in contact-mediated migration^6,8,39^. These features may allow the transplanted OECs to penetrate into the SCI site and integrate following transplantation^6,8^. Additionally, the total membrane protrusion surface area seen in OECs has been shown to increase up to 13 times following GDNF administration, resulting in a contact-mediated migration and the formation of a cellular network^6^. Similarly, at lower concentrations (10 to 20 ng/mL), OECs have been shown to decrease intercellular distance and form tight chains of adherent cells which act as a growth substrate for neurons to follow during regeneration^73^. As OECs are capable of intermingling with reactive astrocytes found in the astrocytic scar, OEC chain formations may allow axonal extension through this otherwise impenetrable environment^3,21^. Furthermore, GDNF is strongly implicated in the survival of dopaminergic and autonomic neurons, where it dramatically increases neurite differentiation and outgrowth at a half-maximal effect concentration of 1.2 pM^38^. Thus, GDNF is unlikely to have detrimental effects on cells endogenous to the injury site.

BDNF is a neurotrophin secreted from OECs, dorsal root ganglion neurons, and myocytes and is a promising neurotrophic agent for SCI therapies when used alone or in conjunction with OEC transplants^25,74,75^. BDNF has been implicated in neuroprotective and morphological changes^76^. For example, BDNF has been shown to stimulate thicker cellular processes of retinal ganglion cells (RGCs) and has greatly increased RGC migration in scratch assays^77^. Another study showed that following transplantation of OECs into a transected optic nerve, the RGC life span was increased, correlating with the concentration of BDNF secreted from OECs. This suggests that the key to increased RGC survival was both the presence of OECs and OEC-secreted BDNF. In the PNS, BDNF secretion can promote migration and myelination by SCs. Signaling through GTPase Ras-related C3 botulinum toxin substrate 1 (Rac1) influence myelination by SCs^25,78^. Increasing levels of active Rac1 are caused by upregulated 3’,5’-cyclic adenosine monophosphate (cAMP) production due to BDNF stimulation. These high levels of Rac1 have been shown to produce radial lamellae in SCs—a phenotype that is highly conducive to myelination and sorting of axons^79^. By increasing cAMP production further through the addition of forskolin (which stimulates cAMP production), it may be possible to amplify the BDNF-induced myelination; however, further investigation into whether these same myelinating effects can be seen in OECs is still required^78,80^.

## Effects of Neurotrophins at the SCI Site Following Transplantation

A continued regimen of neurotrophins, applied postsurgery, could be used to further support the SCI patient during their recovery. In addition to being used during OEC expansion in vitro and as a supportive compound during transplantation, neurotrophins could be used to support the greater injury site area. Neurons, astrocytes, oligodendrocytes, and surrounding cells all express a variety of neurotrophic receptors which may be used to manipulate the SCI site to promote neurogenesis, transplant integration, and angiogenesis.

### Neurons within the SCI Site

As the primary goal of SCI therapies focusses on the regeneration of neurons, neurotrophins that aid the regeneration and migration of neurons are the factors most likely to be beneficial to repair^67,81^. One candidate neurotrophin beneficial to repair might be NT-3. It has been demonstrated that transplanted OECs genetically modified to produce high amounts of NT-3 cause significantly greater axonal regeneration and neuronal sprouting in a thoracic 9 contusion SCI site than control OECs^51^. Furthermore, in RGCs, BDNF has been shown to stimulate thicker cellular processes, greatly increase migration, and extend survival of RGCs following injury suggesting that the same effects may be seen in neurons found in an SCI site^77^. Fibroblast growth factor 2 (FGF2) has been shown to play a role in neuronal maturation which may also be applied to a SCI site for therapeutic benefit. Similarly, FGF2 (25 ng/mL) has been shown to stimulate the maturation of bipolar ganglionic cells from embryonic rat cochlear ganglion tissue^82,83^.

### Astrocytes within the SCI Site

One of the features that makes OECs a valuable transplant candidate for further investigation is that they possess the unique ability to interact with astrocytes while other glial cells do not^3^. This phenotype is attributed to the interaction of FGF2 and heparin molecules on the target cells, which are cell-type specific^82,84^. In contrast, SCs do not naturally interact with astrocytes. However, inhibition of FGFR1, which specifically inhibits the binding of FGF2, leads to an increased mingling of SCs with astrocytes. Additionally, the use of heparinase (a heparin sulfate digestive enzyme) results in the removal of the boundary between the 2 cell types^3,85,86^. The degradation of the boundary between SCs and astrocytes is due to the interaction of FGF with the heparin sulfate proteoglycan profile of which SCs and OECs are different^3^. These results show that while neurotrophins play a beneficial role for 1 cell type, such as promoting survival or migration of OECs, in other circumstances they can be detrimental to the efforts of SCI treatment, for example, by preventing cell integration.

### Oligodendrocytes within the SCI Site

Following SCI, a large number of oligodendrocytes die^49^. Stimulating the survival or proliferation of these cells to support recovering neurons through remyelination and continued survival could be an important step in SCI recovery. It has previously been shown that EGF (20 ng/mL) when applied to injured oligodendrocytes can stimulate the outgrowth of myelinating lamellipodial processes^87^. Interestingly, this feature is not seen in developing or mature oligodendrocytes, only in damaged cells. Conversely, EGF has been demonstrated to suppress the expression of myelin basic protein expression in developing oligodendrocytes and could cause issues with oligodendrocyte precursors found within the SCI site^1,88^.

### Angiogenesis within the SCI Site

As previously discussed, GDNF would be a useful candidate for a coadministrative approach to transplantation. However, GDNF also possesses properties that would be beneficial after the cells have been transplanted, both for the transplanted cells and for the cells at the injury site. The role of angiogenesis in SCI repair is often overlooked; however, the reinnervation of blood supply to regenerating tissue is important for supporting the newly generating neurons and glia. While OECs have previously been shown to encourage angiogenesis^89^, GDNF additionally stimulates an increase in the number of blood vessels compared to a control^19,39^. Similarly, NGF and VEGF demonstrate an ability to stimulate angiogenesis. NGF demonstrates an ability to increase angiogenesis in ischemic hindlimbs^90^, and VEGF can stimulate vascular sprouting in endothelial cells when under hypoxic conditions^91–93^.

A variety of neurotrophins could be applied to the SCI therapy. As autologous OECs constitute a promising therapy option, supporting them following transplantation using NGF, BDNF, NT-3, and GDNF may improve the efficacy of this treatment. Additionally, by influencing cell comingling and angiogenesis using the FGF family of neurotrophins, VEGF, and GDNF, it may be possible to further support the SCI site by improving nutrient innervation within the damaged tissues. If effects of neurotrophins on different cell types in a multicellular environment can be defined, the outcomes may also pave the way for enhancing therapies using other transplanted cell types, such as SCs, oligodendrocytes, and neural stem cells, perhaps in combination with OECs. Neurons, SCs, and oligodendrocytes all express a different profile of neurotrophin receptors as shown in Table 1.

## Future Avenues for Optimization of OEC Transplantation Using Neurotrophins

The neurotrophins discussed in this review and their potential applications in SCI therapies offer an avenue by which the therapeutic potential of transplanted OECs may be significantly enhanced. However, the process of defining a cocktail of neurotrophins to be used to complement OEC transplantation may be hindered by unforeseen interactions between these different proteins. For this reason, the application of neurotrophins in phases may provide the least complications with this treatment. Additionally, application outside of these proposed phases may further aid the patient, for example, as an emergency application following injury or used prior to biopsy so that the greatest yield of functional cells can be achieved.

### Compounding Neurotrophic Effects

As discussed so far, there are several neurotrophins that are applicable in different stages of the transplantation process; however, examining the compounding effects of these different neurotrophins has not been thoroughly investigated. Understanding how neurotrophins interact with each other may provide insight into how to achieve the greatest effect. For example, when exposed to FGF2, platelet-derived growth factor receptor alpha (PDGFRα) expression has been shown to increase. When FGF2 administration was followed with PDGF administration, the resulting increase of mitogenic activity was 30% greater than PDGF alone^94^. Similarly, EGF has shown altered effects when combined with another growth factor. EGF (10 ng/mL) has been shown to promote cell proliferation and differentiation within the basal olfactory neuroepithelium. However, when transforming growth factor beta (TGFβ-2) is used in conjunction with EGF, neuron differentiation is stimulated whereas EGF alone encourages fibroblast differentiation^33,95^. By exploring the use of EGF with varying levels of TGFβ-2, it may be possible to cotransplant OECs and basal cells and then further differentiate neurons and fibroblasts gradually in the days following transplantation, replicating the regenerative abilities of the olfactory system at the level of the cytoenvironment.

**Fig. 4.**
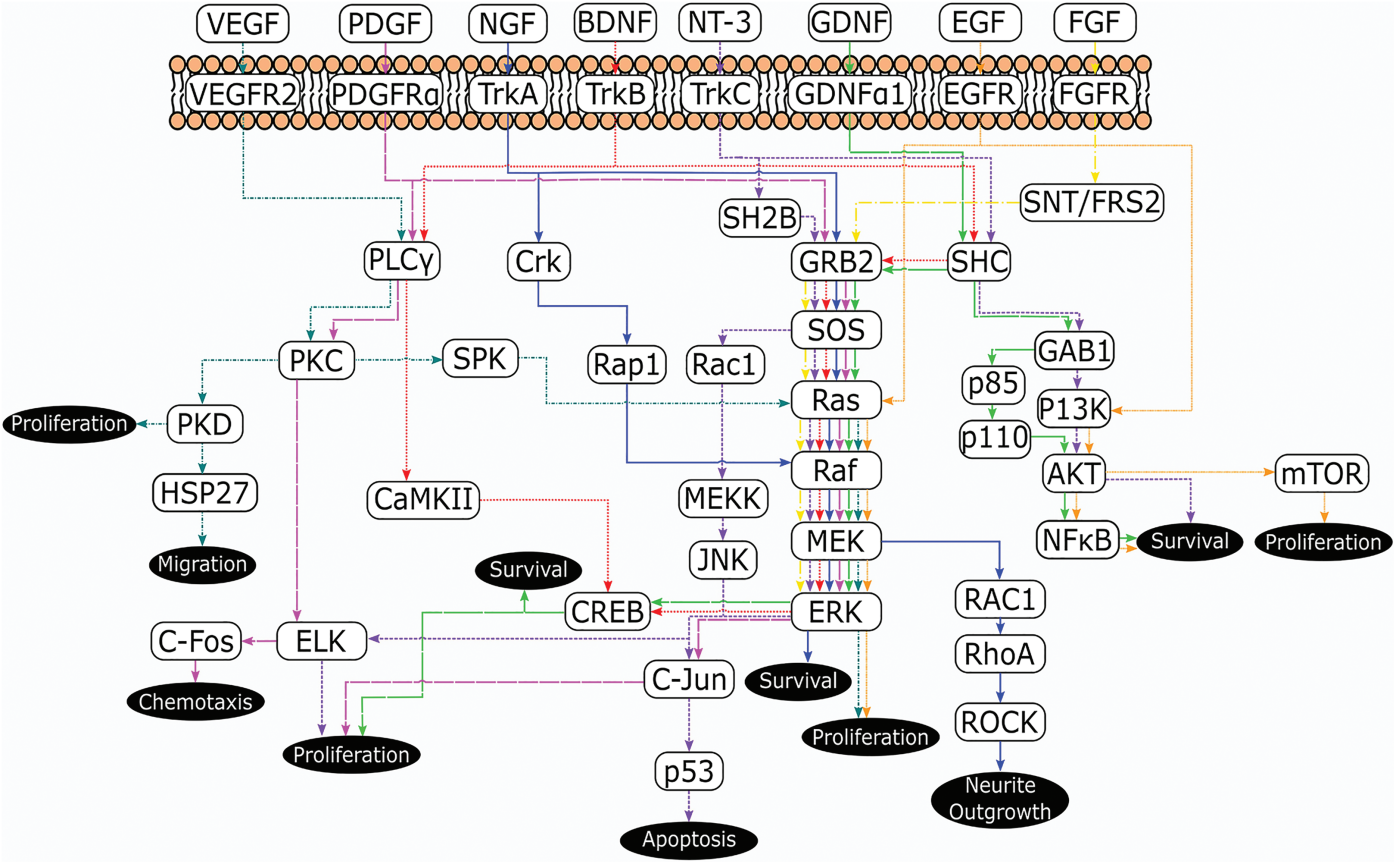
Simplified pathway map of the neurotrophic signaling cascades described in this review and their subsequent effects. *Source*: Adapted from references 32, 52–54, and 96–98.

The modulation of neurotrophic factor receptors by other neurotrophins is an area that requires further research. As many of the target cells possess more than a single neurotrophin receptor, understanding the interplay between these receptors could prove important in the division of an effective treatment. Several of the signaling pathways for different neurotrophins share elements (Fig. 4). In particular, the GRB2-SOS-Ras-Raf-MEK-ERK pathway is shared by all neurotrophins. There are also other similarities such as PDGFRα, VEGFR2, and TrkB signaling via phospholipase C gamma-protein kinase C (PLCγ-PKC), and TrkA, and GFRα1/Ret signaling via RAC1-RhoA. While what is presented in Fig. 4 is a simplified signaling map, it demonstrates the crossover between several neurotrophin signaling cascades and so interaction between these proteins should be further characterized before being applied in future treatments^32,52–54,96–98^.

### Neurotrophins for Emergency Administration

As with many acute injuries, addressing an SCI quickly following injury yields the greatest functional outcome. However, beyond providing therapeutic hypothermia^99^, there is little that can be done to diminish the secondary damage that will occur following SCI. However, neurotrophins may be suited to decreasing this secondary damage when applied acutely. The dual β-subunit of PDGF (PDGF-BB) has been previously used to ameliorate ischemic stroke damage in a dose-dependent manner. Additionally, PDGF-BB has been shown to decrease neuronal death in forebrain ischemia, improving ischemic outcome. While this has not been investigated in the spinal cord, these anti-ischemic properties of PDGF may be translatable to SCI^100^. An interesting combinatorial effect of PDGF with VEGF has been observed for acute SCI, where the combination of the 2 growth factors resulted in a dramatic reduction of secondary degeneration compared to when either growth factor alone was applied to the injury site^101,102^. This combinatorial effect of growth factors highlights the importance of considering multiple outcomes on different cell types when using growth factors.

## Conclusion

**Fig. 5.**
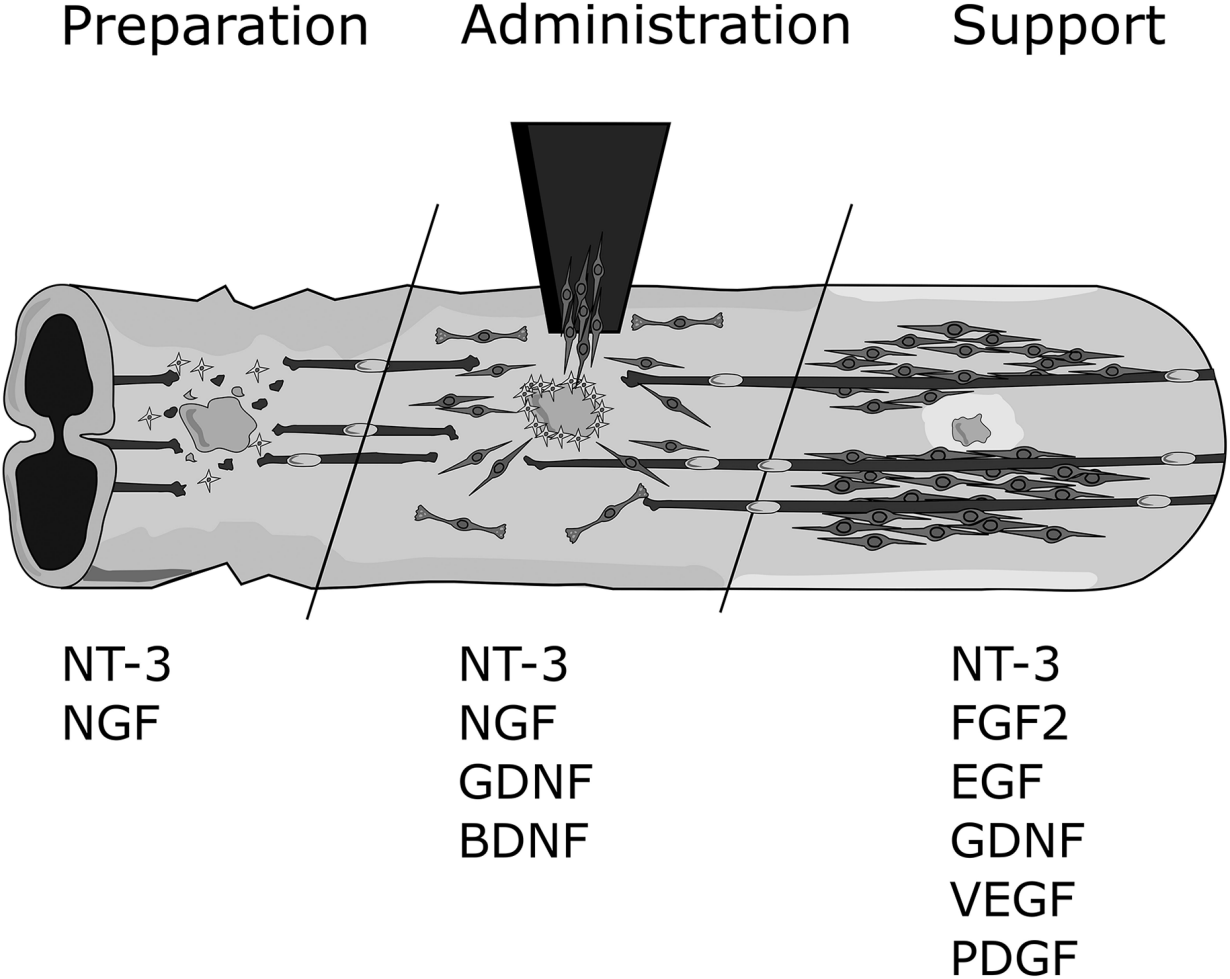
Summary of neurotrophins and their application in spinal cord injury (SCI). The potential roles for key neurotrophins during the 3 phases of olfactory ensheathing cell (OEC) transplantation therapy for SCI (cell preparation, cell administration, and continuous support). OEC preparation may be enhanced using neurotrophin 3 (NT-3) and nerve growth factor (NGF). Cell administration may be supported using NT-3, NGF, glial-derived neurotrophic factor (GDNF), and brain-derived neurotrophic factor (BDNF). Ongoing support to the injury/transplantation site may be achieved through the use of NT-3, fibroblast growth factor 2 (FGF2), epidermal growth factor (EGF), GDNF, vascular endothelial growth factor (VEGF), and platelet-derived growth factor (PDGF).

OEC transplantation is a very promising therapy for SCI; however, results are highly variable and the method needs improvement. Neurotrophins can stimulate favorable behaviors in OECs, such as proliferation, migration, phagocytosis, and interaction with other cells. Prior to transplantation, neurotrophins can be used to optimize the health, quality, and purity of OECs. Coadministration of neurotrophins with OECs at the time of transplantation may also be beneficial; here, the neuroprotective factors NGF and NT-3 are likely candidates. GDNF can also be used in this stage of treatment, by aiding the migration and integration of OECs into the SCI site. Finally, neurotrophins may be used as general support for the SCI environment in the longer term. NT-3 (neuroprotection), BDNF (neuronal survival), FGF2 (astrocyte–OEC integration), NGF, GDNF, VEGF, and PDGF (vascularization) are all promising candidates for this purpose (Fig. 5).

Using neurotrophin manipulation, it may be possible to adjust the phenotype and behaviors of OEC into those most beneficial for SCI repair. Determining how neurotrophins modulate OECs and cell types endogenous to the injury site is therefore highly warranted.
